# Granulocytes in cerebrospinal fluid of adults suspected of a central nervous system infection: a prospective study of diagnostic accuracy

**DOI:** 10.1007/s15010-024-02200-5

**Published:** 2024-03-23

**Authors:** Steven L. Staal, Sabine E. Olie, Liora ter Horst, Ingeborg E. van Zeggeren, Diederik van de Beek, Matthijs C. Brouwer, I.-PACE Study Group

**Affiliations:** Amsterdam UMC, Department of Neurology, Amsterdam Neuroscience, University of Amsterdam, Meibergdreef 9, 1105 AZ Amsterdam, the Netherlands

**Keywords:** Central nervous system infection, Bacterial meningitis, Cerebrospinal fluid, Granulocytes, Diagnostic accuracy

## Abstract

**Purpose:**

Cerebrospinal fluid (CSF) granulocytes are associated with bacterial meningitis, but information on its diagnostic value is limited and primarily based on retrospective studies. Therefore, we assessed the diagnostic accuracy of CSF granulocytes.

**Methods:**

We analyzed CSF granulocytes (index test) from all consecutive patients in two prospective cohort studies in the Netherlands. Both studies included patients ≥ 16 years, suspected of a central nervous system (CNS) infection, who underwent a diagnostic lumbar puncture. All episodes with elevated CSF leukocytes (≥ 5 cells per mm^3^) were selected and categorized by clinical diagnosis (reference standard).

**Results:**

Of 1261 episodes, 625 (50%) had elevated CSF leukocytes and 541 (87%) were included. 117 of 541 (22%) were diagnosed with bacterial meningitis, 144 (27%) with viral meningoencephalitis, 49 (9%) with other CNS infections, 76 (14%) with CNS autoimmune disorders, 93 (17%) with other neurological diseases and 62 (11%) with systemic diseases. The area under the curve to discriminate bacterial meningitis from other diagnoses was 0.97 (95% confidence interval [CI] 0.95–0.98) for CSF granulocyte count and 0.93 (95% CI 0.91–0.96) for CSF granulocyte percentage. CSF granulocyte predominance occurred in all diagnostic categories. A cutoff at 50% CSF granulocytes gave a sensitivity of 94% (95% CI 90–98), specificity of 80% (95% CI 76–84), negative predictive value of 98% (95% CI 97–99) and positive predictive value of 57% (95% CI 52–62).

**Conclusion:**

CSF granulocytes have a high diagnostic accuracy for bacterial meningitis in patients suspected of a CNS infection. CSF granulocyte predominance occurred in all diagnostic categories, limiting its value in clinical practice.

## Background

Central nervous system (CNS) infections are a global health problem with high mortality and morbidity rates [1]. Diagnosing CNS infections can pose a challenge as clinical features, such as the classic triad of bacterial meningitis (fever, headache and neck stiffness), lack adequate sensitivity and correspond to a broad differential diagnosis ranging from benign to life-threatening conditions [2]. As clinical features lack diagnostic accuracy, cerebrospinal fluid (CSF) examination is required in the diagnostic work-up of patients suspected of a CNS infection. Conventional CSF examination consists of determining the number of leukocytes and their differentiation, glucose and total protein level, after which microbiological testing is done to identify a pathogen. However, in acute care settings, physicians frequently have to establish a treatment strategy based on the initial CSF results as microbiological testing requires more time. Several prediction models have been established to aid the probability assessment of bacterial meningitis in patients suspected of a CNS infection, often taking into account the relative amount or absolute number of granulocytes [3]. Granulocytes may give information on the type of immune response and an abundance relative to the number of mononuclear cells has been associated with bacterial meningitis [4]. However, little is known about the diagnostic accuracy of CSF granulocytes as most of the current knowledge on CSF leukocyte subsets is based on studies that are limited by a retrospective study design, comparing cases of bacterial meningitis to other pre-defined diagnoses instead of evaluating them in the context of all cases suspected of a CNS infection [5]. Therefore, we evaluated the diagnostic accuracy of CSF granulocytes in patients with a suspected CNS infection.

## Methods

### Study population and data collection

We analyzed CSF granulocyte counts and proportions (= index tests) from all consecutive patients in two prospective cohort studies in the Netherlands. One single-center pilot study (pediatric and adult causes of encephalitis and meningitis [PACEM]) [2] and one ongoing multicenter cohort study (improving prognosis using innovative methods to diagnose causes of encephalitis [I-PACE]) [6], respectively, from the periods 2012 to 2015 and 2017 to now. Both studies included patients ≥ 16 years that were suspected of a CNS infection and who underwent a diagnostic lumbar puncture. Patients were included either at presentation at the emergency department or during admission in case the lumbar puncture was performed at a later time point. Exclusion criteria were a suspected CNS infection within 3 months after neurosurgery and/or a traumatic brain injury or the presence of a neurosurgical or neurostimulatory device within the central nervous system. Eligible patients were reported to the investigators by the treating physician or identified during morning rounds and in laboratory records. Written informed consent was obtained from all participants, or their legal representative when the patient was incapacitated at the time of inclusion. Repeated inclusions of the same patient during the study period were considered only in those who underwent a lumbar puncture for a new suspicion of a CNS infection. Follow-up lumbar punctures of an initial suspicion were not included in the current study. Clinical data were obtained from the patients’ electronic health records and stored in secured online case record forms. All patient data were anonymized in accordance with Dutch privacy legislation.

We selected episodes with abnormal CSF leukocyte counts (i.e., ≥ 5 cells per mm^3^) in which CSF granulocytes were determined as part of routine clinical care and granulocyte predominance was defined as a granulocyte percentage of > 50%. Cell counts and differentiation were conducted using an automated cell counter. Episodes in which CSF granulocytes were not determined were excluded from the analysis. The diagnostic accuracy of CSF granulocytes, as well as CSF leukocytes, was determined. The following measures of diagnostic accuracy were calculated: sensitivity, specificity, predictive values and the area under the curve (AUC). In addition, the Youden’s index, a cutoff value that maximizes the sensitivity and specificity, was determined and evaluated.

Episodes were considered hospital-acquired if they occurred during admission (> 48 h after presentation) or within 1 week after discharge from a previous hospital admission. All other episodes were considered community-acquired. An immunocompromised state was defined as the use of immunosuppressive drugs or a medical history of human immunodeficiency virus (HIV) infection, cancer, diabetes mellitus or alcoholism. The Glasgow Coma Scale (GCS) score was used to describe the level of consciousness at presentation [7]. A GCS-score of ≤ 14 was considered as an altered mental status and a GCS-score of ≤ 8 indicated a coma.

### Diagnostic categories

The final clinical diagnoses (= reference standard) were classified into the following categories, as previously described [2]: CNS infection, CNS autoimmune disorder, other neurological disease and systemic disease; CNS infections were subdivided into bacterial meningitis, viral meningoencephalitis or other CNS infection. All episodes were independently evaluated and classified by two clinicians (SS, SO) and disagreements were resolved in consensus with a third clinician (MB). Cohen’s Kappa coefficient (κ), a measure of inter-observer agreement, was calculated with a κ = 0.76 for the first cohort and κ = 0.58 for the second cohort.

### Statistical analysis

Data analyses and visualization were performed using RStudio version 4.2.1 (RStudio Inc. Boston, USA) and GraphPad Prism version 9.5.1 (GraphPad Software Inc. California, USA). The following additional R packages were used: tidyverse, ggplot2 and pROC. Tidyverse was used for data handling, ggplot2 for data visualization and pROC to produce receiver operator characteristics (ROC) curves and to calculate the area under the curve (AUC), sensitivity, specificity, positive predictive value (PPV) and negative predictive value (NPV). P-values ≤ 0.05 were considered statistically significant.

## Results

### Patient characteristics

Overall, 1261 episodes of suspected CNS infections were included in this study of which 636 episodes (50%) were excluded because of a CSF leukocyte count < 5 cells per mm^3^. Of the remaining episodes, 84 were excluded due to the absence of CSF granulocyte counts. In total, 541 of 1261 episodes were eligible for analysis. The median age of all episodes was 53 years (interquartile range [IQR] 37–66) and 237 of 541 (44%) episodes occurred in females (Table 1). In 38 of 380 (10%) episodes, a previous CNS infection was reported and in 37 of 538 (7%) the episode was defined as hospital-acquired. An immunocompromised state was present in 241 of 541 episodes (45%) and was due to immunosuppressive therapy in 84 of 539 episodes (16%), diabetes mellitus in 84 of 541 episodes (16%), cancer in 75 of 539 episodes (14%) and HIV in 44 of 541 episodes (8%). Upon presentation, headache was the most common symptom occurring in 327 of 472 episodes (69%), followed by nausea or vomiting in 199 of 469 episodes (42%) and seizures in 62 of 513 episodes (12%). Fever was present in 216 of 532 episodes (41%), neck stiffness occurred in 126 of 533 episodes (24%) and an altered mental status in 256 of 538 episodes (48%).

**Table 1:** Baseline characteristics from all eligible episodes^a^

	n/N (%)		n/N (%)
Patient characteristics		Physical examination	
Age, years	53 (37–66)	Temperature, °C^b^	38 (37–39)
Sex, female	237/541 (44)	Fever, ≥ 38 °C	216/532 (41)
Previous central nervous system infection	38/380 (10)	Hypotension	13/528 (3)
Suspected hospital-acquired infection	37/538 (7)	Tachycardia	41/527 (8)
		Tachypnea	97/333 (29)
Immunocompromised state	241/541 (45)		
Immunosuppressive therapy	84/539 (16)	Neurological examination	
Diabetes mellitus	84/541 (16)	Neck stiffness	126/533 (24)
Cancer	75/539 (14)	Glasgow Coma Scale score^c^	15 (12–15)
Known HIV-positive	44/541 (8)	Altered mental status, GCS ≤ 14	256/538 (48)
Alcoholism	31/473 (7)	Coma, GCS ≤ 8	52/538 (10)
		Aphasia	54/540 (10)
Presenting symptoms		Papilledema	10/539 (2)
Headache	327/472 (69)	Ataxia	19/541 (4)
Vomiting or nausea	199/469 (42)	Babinski reflex	46/537 (9)
Seizures	62/513 (12)	Cranial nerve palsy present	78/539 (15)
		Motor abnormalities present	71/540 (13)

^a^Data are shown as frequency (%) or median (interquartile range [IQR]). Human immunodeficiency virus (HIV)

^b^Temperature was recorded for 532 episodes

^c^Glasgow Coma Scale (GCS) score was recorded for 538 episodes

### Final diagnoses

**Fig. 1:**
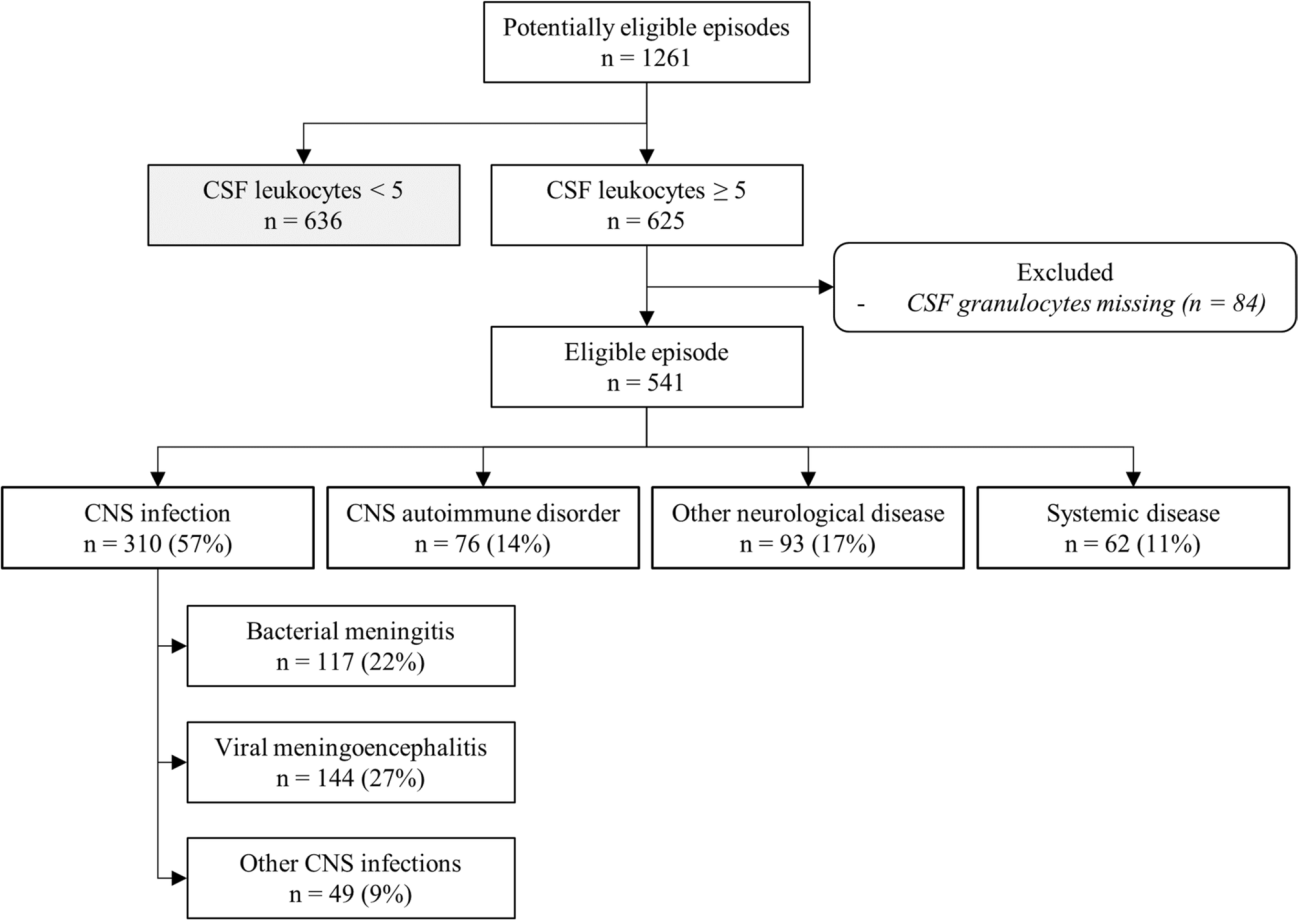
Flowchart of episodes through the study. *Cerebrospinal fluid (CSF), central nervous system (CNS)*

A CNS infection was diagnosed in 310 of 541 episodes (57%; Fig. 1). Of those, viral meningoencephalitis was the most common cause and occurred in 144 of 541 episodes (27%). Bacterial meningitis and other CNS infections occurred in, respectively, 117 (22%) and 49 (9%) of 541 episodes. The remaining episodes were diagnosed as CNS autoimmune disorder in 76 of 541 episodes (14%), other neurological disease in 93 of 541 episodes (17%) or systemic disease in 62 of 541 episodes (11%).

### Cerebrospinal fluid characteristics

**Fig. 2:**
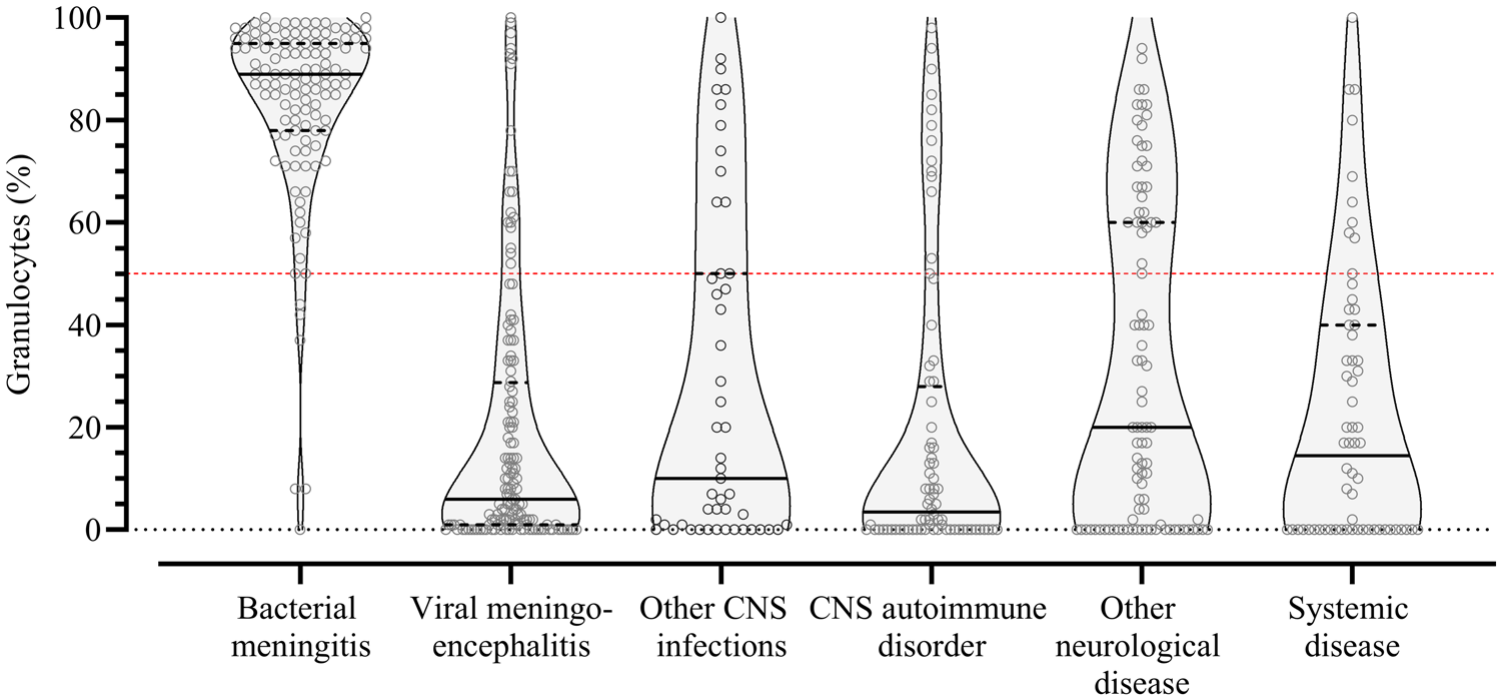
Violin plots depicting CSF granulocyte percentage per diagnostic category. Red dashed line represents a cerebrospinal fluid granulocytes percentage of 50%. Patients above demonstrated granulocyte predominance. Cerebrospinal fluid (CSF), central nervous system (CNS)

The median CSF leukocyte count of all episodes suspected of a CNS infection was 60 per mm^3^ (IQR 11–389), with a median CSF granulocyte count of 5 per mm^3^ (IQR 1–123). The median granulocyte percentage was 20% (IQR 1–73) and 191 of 541 episodes (35%) had granulocyte predominance (Table 2). Episodes diagnosed with bacterial meningitis had a median CSF leukocyte count of 2189 cells per mm^3^ (IQR 876–6670) with a median granulocyte count of 1571 cells per mm^3^ (IQR 658–6211). The median granulocyte percentage was 89% (IQR 78–95) and 109 of 117 episodes (93%) demonstrated granulocyte predominance (Table 2 and Fig. 2). The median CSF leukocyte count of episodes diagnosed with viral meningoencephalitis was 101 cells per mm^3^ (IQR 34–237) with a median granulocyte count of 5 cells per mm^3^ (IQR 1–23). The median granulocyte percentage was 6% (IQR 1–28) with a granulocyte predominance in 20 of 144 episodes (14%). Episodes with other CNS infections had a median CSF leukocyte count of 95 (IQR 30–319) and a median granulocyte count and percentage of 4 cells per mm^3^ (IQR 1–106) and 10% (IQR 0–50), respectively. In 11 of the 49 episodes (22%) with other CNS infections, the CSF demonstrated granulocyte predominance. Granulocyte predominance occurred in all diagnostic categories (Fig. 2). Eight of 117 episodes (7%) diagnosed with bacterial meningitis had a granulocyte percentage of 50% or lower. Of those, four had a concomitant positive culture of either CSF (*Streptococcus pneumoniae*, *Listeria monocytogenes* or *Rhodotorula mucilaginosa*) or blood (*Streptococcus bovis*). The remaining four episodes were CSF or blood culture negative.

**Table 2:** CSF characteristics per diagnostic category^a^

		CNS infections					
	Overall	Bacterial meningitis	Viral meningoencephalitis	Other CNS infections	CNS autoimmune disorder	Other neurological disease	Systemic disease
	(*N* = 541)	(*N* = 117)	(*N* = 144)	(*N* = 49)	(*N* = 76)	(*N* = 93)	(*N* = 62)
Leucocytes, cells per mm^3^	60 (11–389)	2189 (876–6670)	101 (34–237)	95 (30–319)	34 (12–89)	10 (6–23)	6 (5–10)
Granulocytes, cells per mm^3^	5 (1–123)	1571 (658–6211)	5 (1–23)	4 (1–106)	1 (0–7)	2 (0–6)	1 (0–3)
Granulocytes, percentage	20 (1–73)	89 (78–95)	6 (1–28)	10 (0–50)	3 (0–26)	20 (0–60)	15 (0–39)
Granulocyte, > 50% (%)	191 (35)	109 (93)	20 (14)	11 (22)	12 (16)	30 (32)	9 (15)
CSF to blood glucose ratio^b^	0.51 (0.37–0.60)	0.14 (0.01–0.36)	0.54 (0.49–0.61)	0.45 (0.36–0.56)	0.53 (0.46–0.60)	0.54 (0.46–0.65)	0.59 (0.53–0.64)
Protein, g/L^c^	0.79 (0.48–1.63)	3.02 (1.48–4.89)	0.68 (0.48–1.02)	0.88 (0.56–1.29)	0.72 (0.48–1.01)	0.56 (0.37–0.94)	0.46 (0.35–0.65)

^a^Data are shown as median (interquartile range [IQR]) or n (%). Cerebrospinal fluid (CSF), central nervous system (CNS)

^b^CSF to blood glucose ratio was recorded for 480 episodes (110 episodes with bacterial meningitis, 131 episodes with viral meningoencephalitis, 41 episodes with other CNS infections, 60 episodes with CNS autoimmune disorder, 82 episodes with other neurological disease and 56 episodes with systemic disease)

^c^Protein level was recorded for 537 episodes (116 episodes with bacterial meningitis, 143 episodes with viral meningoencephalitis, 48 with other CNS infection, and 61 with systemic disease)

### Diagnostic accuracy

**Fig. 3:**
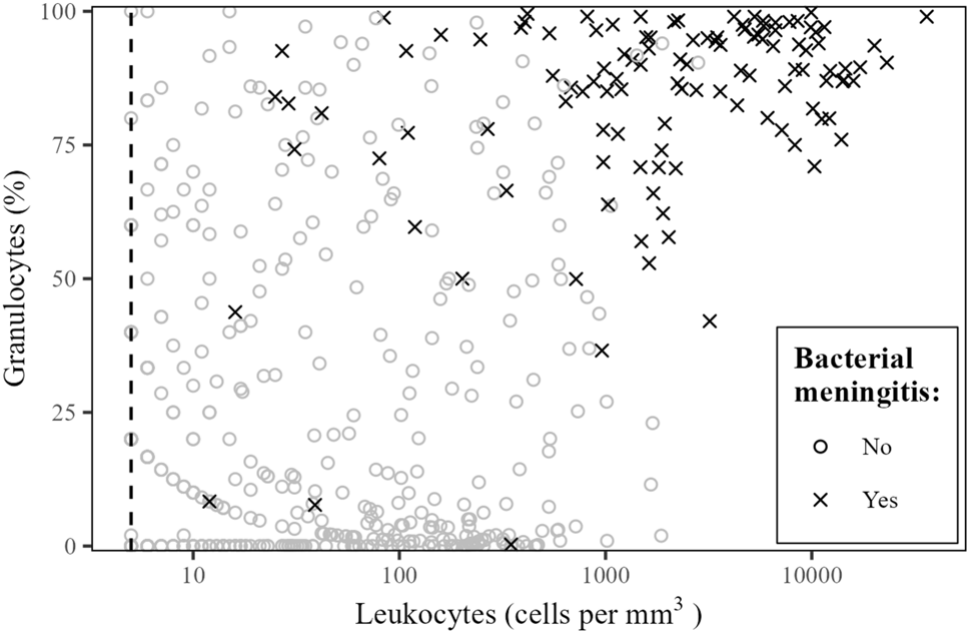
Scatterplot depicting granulocyte percentage in relation to leukocyte count in the CSF. Vertical dashed line represents a cerebrospinal fluid leukocyte count of 5. Crosses represent patients diagnosed with bacterial meningitis. Cerebrospinal fluid (CSF)

**Fig. 4:**
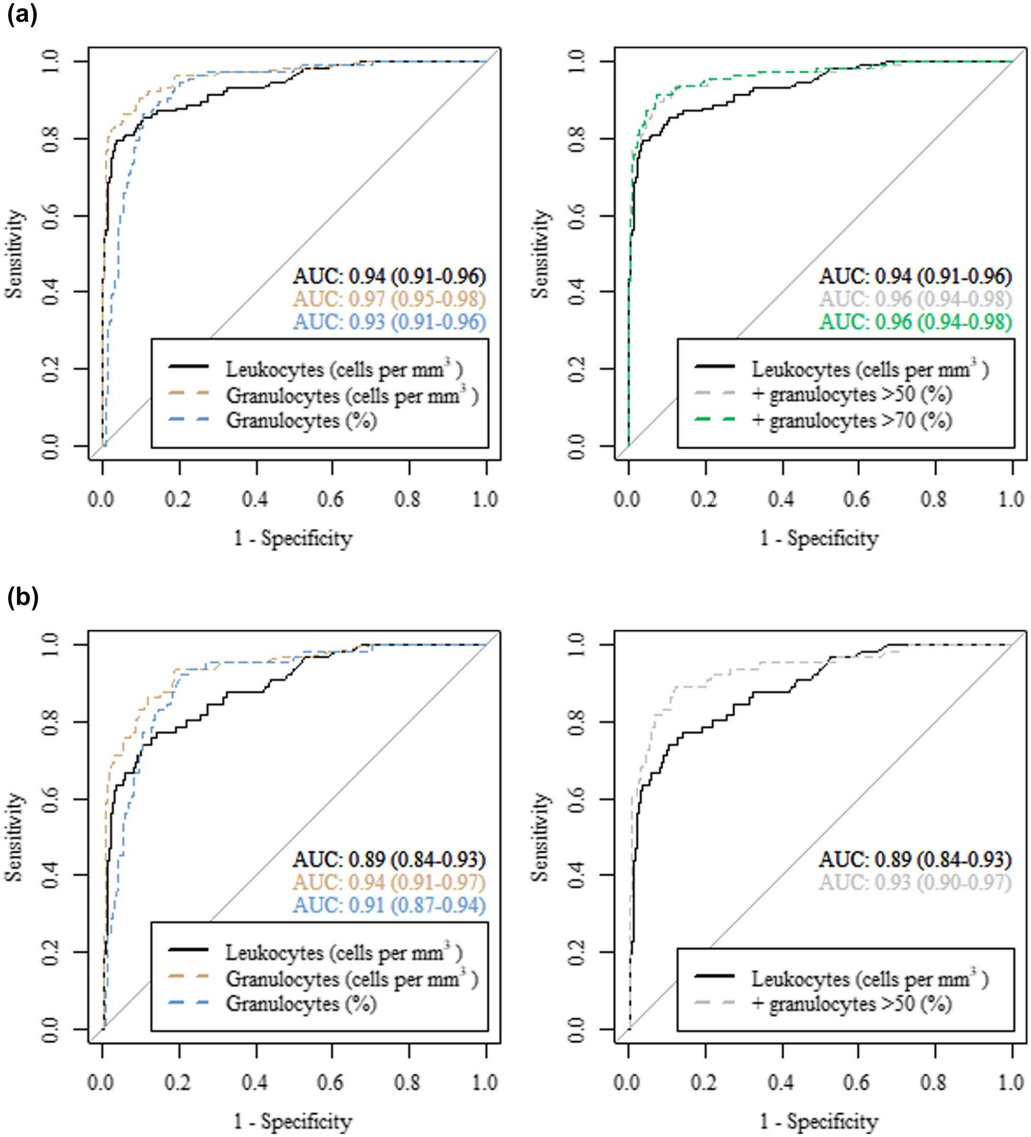
**a** ROC curves of leukocytes and granulocytes in the CSF of all episodes. **b** ROC curves of leukocytes and granulocytes in the CSF of episodes with 5–3000/mm^3^ CSF leukocytes. The AUCs of leukocytes, granulocytes and granulocyte percentage in the CSF as a single predictor for bacterial meningitis are depicted in, respectively, black, yellow and blue. The AUCs of a logistic regression model using bacterial meningitis as the dependent variable and leukocytes together with a cutoff point for granulocyte percentage at either granulocyte predominance (> 50%) or the Youden index as the independent variables are depicted in, respectively, in grey and green. Receiver operator characteristics (ROC), cerebrospinal fluid (CSF), area under the curve (AUC).

The majority of episodes diagnosed with bacterial meningitis were characterized by high leukocyte counts and a high granulocyte percentage in the CSF (Fig. 3). A specificity of 100% for the diagnosis of bacterial meningitis was established at a cutoff point of 2967 CSF leukocytes per mm^3^ and 2754 CSF granulocytes per mm^3^ with a similar sensitivity of, respectively, 44% (95% confidence interval [CI] 34–52) and 43% (95% CI 34–51) (Table 3). A cutoff at 50% CSF granulocytes showed a sensitivity of 94% (95% CI 90–98) and specificity of 80% (95% CI 76–84). The Youden index for granulocyte percentages (70% CSF granulocytes) showed a sensitivity and specificity of, respectively, 86% (95% CI 80–92) and 90% (95% CI 87–92). The AUC for predicting bacterial meningitis using a single predictor was 0.94 (95% CI 0.91–0.96) for CSF leukocytes, 0.97 (95% CI 0.95–0.98) for CSF granulocytes per mm^3^ and 0.93 (95% CI 0.91–0.96) for CSF granulocyte percentage (Fig. 4).

**Table 3:** Test characteristics of CSF leukocytes, granulocytes and granulocyte percentage for predicting bacterial meningitis in patients suspected of a CNS infection

		Cutoff point	Sens	(95% CI)	Spec	(95% CI)	NPV	(95% CI)	PPV	(95% CI)
a. All episodes suspected of a CNS infection with elevated CSF leukocytes										
Leukocytes, cells per mm^3^	Youden index	635	79	(72–87)	96	(95–98)	94	(93–96)	86	(80–92)
	100% sensitivity	12	100	–	32	(28–37)	100	–	29	(28–30)
	100% specificity	2967	44	(35–52)	100	–	87	(85–88)	100	–
Granulocytes, cells per mm^3^	Youden index	82	91	(85–96)	90	(88–93)	97	(96–99)	72	(66–78)
	100% sensitivity	1	100	–	30	(25–34)	100	-	28	(27–29)
	100% specificity	2754	43	(34–51)	100	–	86	(85–88)	100	–
Granulocytes, percentage	Youden index	70	86	(80–92)	90	(87–92)	96	(94–98)	70	(64–76)
	Predominance	50	94	(90–98)	80	(76–84)	98	(97–99)	57	(52–62)
b. All episodes suspected of a CNS infection with CSF leukocytes between 5 and 3000 cells per mm^3^										
Leukocytes, cells per mm^3^	Youden index	331	74	(64–85)	89	(87–92)	96	(94–97)	52	(45–60)
Granulocytes, cells per mm^3^	Youden index	20	94	(88–98)	81	(77–85)	99	(98–100)	43	(39–49)
Granulocytes, percentage	Youden index	50	92	(85–98)	80	(76–83)	99	(97–100)	42	(37–47)
	Predominance	50	91	(83–97)	80	(76–84)	98	(97–99)	41	(37–47)

Cerebrospinal fluid (CSF), central nervous system (CNS), sensitivity (Sens), specificity (Spec), positive predictive value (PPV), negative predictive value (NPV)

A logistic regression model was fitted to predict the probability of being diagnosed with bacterial meningitis using a combination of CSF leukocytes with a cutoff at either 50% or 70% (Youden index) CSF granulocytes. The model using a cutoff of 50% CSF granulocytes yielded an AUC of 0.96 (CI 0.94–0.98) and the model using a cutoff of 70% CSF granulocytes yielded an AUC of 0.96 (CI 0.94–0.98). Since a CSF leukocyte count of 2967 cells per mm^3^ ruled out other diagnoses (positive predictive value, PPV 100%), the same test characteristics were calculated for a subgroup of episodes with leukocyte counts between 5 and 3000 cells per mm^3^ (approximation of 2967 mm^3^) which showed a similar outcome (Table 3 and Fig. 4).

## Discussion

In this prospective study of diagnostic accuracy, we showed that CSF granulocytes have a high diagnostic accuracy for diagnosing bacterial meningitis among patients suspected of a CNS infection. The association between bacterial meningitis and elevated CSF granulocytes has been a consistent finding [4]. CSF granulocyte predominance was found mostly in episodes with bacterial meningitis as well; however, its occurrence was evident across all diagnostic categories limiting its utility in providing guidance for therapeutic considerations. These results correspond with the findings on CSF granulocyte percentage in two large retrospective cohort studies [8, 9]. Both studies, containing CSF samples of > 1000 patients with elevated CSF leukocytes, described the causes of CSF pleocytosis, their corresponding CSF features and the predictive value of CSF leukocytes (alone or as part of the CHANCE score) for the diagnosis of bacterial meningitis. Although the predictive value of CSF leukocytes alone was similar to the one found in our study, none of the studies mentioned the predictive value of CSF granulocytes. In addition, both studies were limited by a retrospective study design in which cases of bacterial meningitis were compared to other cases with a pre-defined diagnosis instead of all cases suspected of a CNS infection. Comparisons as such do not render reliable predictive values for the diagnosis of bacterial meningitis, as part of the study population may not have had an initial suspicion of a CNS infection. Whereas the sensitivity of CSF features derived from those studies might be similar in the context of patients suspected of a CNS infection, its specificity will likely be lower.

CSF granulocyte predominance in viral CNS infections has been described before, for instance a prospective cohort study of 418 patients with enterovirus meningitis found that 28% presented with granulocyte predominance, especially in the early days of the disease [10]. Conversely, in bacterial meningitis, CSF granulocyte predominance may be absent. The occurrence of *Listeria monocytogenes* in those episodes was expected as lower CSF granulocyte proportions have already been described [11] and granulocyte predominance is found in only 65–81% of cases [12]. However, also for *Streptococcus pneumoniae*, the most common cause of bacterial meningitis [13] which typically manifests with a high leukocyte count in CSF [14], we found a case with monocyte predominance. Lower CSF leukocyte counts in pneumococcal meningitis (less than 1000 cells per mm^3^) have been described to occur in 33% of cases [14, 15] and even cases with normal CSF leukocyte counts have been described in 2% [16]. However, in those instances, the granulocyte proportions are often not determined and CSF leukocyte differentiation is not performed when the leukocyte count is normal. Despite its high diagnostic accuracy in bacterial meningitis, CSF granulocytes do not discriminate perfectly. This finding stresses the need for new diagnostics to differentiate CNS infections or discriminate CNS infections from non-CNS infections, especially in the acute care setting when microbiological examination is still pending. Measurements of inflammatory proteins in CSF, such as C-reactive protein (CRP) and cytokines, may be an interesting option to explore as their measuring techniques are already implemented in many clinical laboratories and results are rapidly available. Several studies have evaluated the diagnostic accuracy of various inflammatory markers in CSF to identify patients with bacterial meningitis [17–20]. Some CSF markers, such as interleukin (IL-)6, IL-10, CRP and procalcitonin, show considerably discriminatory capabilities but do not outperform routinely measured CSF parameters [17–20]. These findings are derived from small study populations that do not accurately represent clinical practice. Larger studies on diagnostic accuracy that include all patients suspected of a CNS infection are needed to evaluate the added diagnostic value of either individual or combinations of inflammatory markers in CSF.

Our study is subject to certain limitations. First, 636 of 1261 episodes (50%) had a normal CSF leukocyte count (< 5 leukocytes per mm^3^) and were filtered out due to the absence of information on leukocyte determination in the majority of cases as this was frequently omitted in the diagnostic work-up. Although this comprises half of the potentially eligible episodes, it does not reduce the validity of this study as those episodes would no longer be suspected of a CNS infection. Second, the Cohen’s Kappa coefficient ranges from moderate to substantial, demonstrating a considerable amount of disagreement between the observers when evaluating the episodes. Even though this may have led to the misclassification of some episodes, a certain amount of inter-observer disagreement was to be expected in a study population of this size and complexity. Furthermore, it underlines the importance of novel diagnostic strategies to differentiate patients with a higher degree of certainty. Finally, a substantial number of episodes were included in a tertiary referral center with specific expertise in CNS infections. This probably introduced selection bias as episodes may have been included, for instance, during a hospital admission in the context of a second opinion. However, the majority of patients came from the region in which the hospital was located and was thus an approximation of the average population. Although this might have a slight effect on the external validity of the results, it does provide relevant information about atypical or complex cases.

## Conclusion

CSF granulocytes have a high diagnostic accuracy to diagnose bacterial meningitis among patients suspected of a CNS infection. However, CSF granulocyte predominance occurred in all diagnostic categories, limiting its use in clinical decision making. Instead, it is safer to base treatment decisions on microbiological examination, which is indicated regardless of the CSF leukocyte count or its differentiation.

## Data Availability

Data protection regulations in the Netherlands do not allow for sharing of individual participant data. Datasets with selected aggregated data is available upon request. Proposals can be directed to ipace@amsterdamumc.nl.
